# COVID-19 vaccine hesitancy and GAD: The role of risk perception and vaccination status

**DOI:** 10.3389/fpubh.2022.994330

**Published:** 2022-10-19

**Authors:** Bin Wang, Xiao Zhong, Haojie Fu, Mengting He, Ruilin Hu

**Affiliations:** ^1^Psychosocial Service and Crisis Intervention Research Center, Southwest University of Science and Technology, Mianyang, China; ^2^School of Humanities and Social Science, University of Science and Technology of China, Hefei, China; ^3^Department of Psychology, Beijing Sport University, Beijing, China; ^4^Shanghai Research Institute for Intelligent Autonomous Systems, Tongji University, Shanghai, China

**Keywords:** COVID-19, vaccine hesitancy, generalized anxiety disorder, risk perception, vaccination status

## Abstract

**Background and purpose:**

As Coronavirus disease (COVID-19) continues to spread around the world, COVID-19 vaccines are the most effective weapons against the global pandemic. Yet vaccine hesitancy remains a serious problem and can pose certain hazards to individuals' mental health, such as rising anxiety. Therefore, based on Self-Discrepancy Theory, this paper aims to explore the role of COVID-19 vaccine hesitancy on individual generalized anxiety disorder and its influence mechanisms through two studies.

**Methods:**

Study one involved 654 Chinese participants using the Vaccine Hesitancy Questionnaire and the Generalized Anxiety Disorder 7-item (GAD-7) scale. In Study two, the Vaccine Hesitation Questionnaire, GAD-7 scale, Perceived Risk of COVID-19 pandemic scale, and Vaccination Status Questionnaire were used and data from 3,282 Chinese residents was collected.

**Results:**

Vaccine hesitancy directly increases generalized anxiety disorder; risk perception plays a partial mediating role between vaccine hesitancy and generalized anxiety disorder; vaccination status moderated vaccine hesitancy's effect on risk perception and generalized anxiety disorder.

**Conclusion:**

Vaccine hesitancy predicts generalized anxiety disorder through risk perception, but the mediating role of risk perception is moderated by vaccination status, which means that for the vaccinated group when their vaccine hesitancy is reduced, it will be easier to reduce the risk perception and thus the generalized anxiety disorder.

## Introduction

The Coronavirus disease (COVID-19) has wreaked great havoc on our society. Until a drug with proven efficacy against this virus is found, COVID-19 vaccines are considered to be the most effective means of controlling this contagion (1), offering a ray of hope for individuals and societies suffering from the pandemic (2). Although several available COVID-19 vaccines have been developed, the current situation of vaccination is not promising (3). The prevalent phenomenon of vaccine hesitancy (VH) undermines global efforts to combat the COVID-19 pandemic (4) and has a negative impact on the psychological state of individuals, bringing about fear and anxiety (5). VH refers to a delay in acceptance or refusal of vaccination despite the availability of vaccination services (6). It is an increasingly prominent problem and is now listed by the World Health Organization (7) as one of the greatest threats to global health. From the perspective of technophobia (8), VH is always accompanied by the emergence of epidemics and the development of vaccine technology. We need to focus not only on the causes of VH but more importantly, on the risks associated with VH. Therefore, this study explores the psychological risks associated with VH from a psychological or psychiatric perspective.

### The role of VH in GAD

The public was generally in a state of anxiety during COVID-19 and even suffer from Generalized Anxiety Disorder (GAD) (9, 10), which is characterized by excessive, persistent, and unrealistic worry about everyday things. The epidemiological and social crisis brought about by COVID-19 has amplified the existing social anxiety (11). Does VH also amplify anxiety as a risk factor for GAD? Higgins' Self-Discrepancy Theory proposes that individuals engage in self-evaluation in three domains: the actual self, the ideal self, and the ought self (12). The actual self is what the individual believes he or she is; the ideal self is what the individual would like to be, and the ought self is what the individual believes he or she should be about responsibilities and obligations. Self-Discrepancy Theory focuses on two types of a discrepancy, namely, the discrepancy between the actual self and ideal self, and the discrepancy between the actual self and ought self (13). And actual self-should self-discrepancy is uniquely related to anxious depressive mood (14).

As universal individual decision-making and the cognitive phenomenon, hesitancy is mainly manifested by individuals' hesitation to choose among multiple options or their inability to decide promptly (15). And VH occurs on the continuum between high vaccine demand and complete vaccine refusal (16). When making decisions, the reality of inadequate information for decision-making creates difficulties for decision-makers to anticipate possible outcomes and the probability of each outcome promptly, and decision-makers are prone to hesitate by repeatedly questioning the correctness of the decisions made. Hesitation reflects the actual self-should self-discrepancy, i.e., the discrepancy between the consideration of the efficacy and risk of a vaccine (actual self) and the responsibility for inoculation (should self). This can lead to adverse psychiatric symptoms such as anxiety in individuals (17). A study also demonstrated a high correlation between VH and anxiety related to Coronavirus (5). Based on this, the following hypothesis is proposed in this paper.

**H1:** VH is positively predictive of GAD.

### The mediating role of RP

VH also reflects uncertainty about emerging technologies (11), which arises when it is difficult for the public to predict precisely the probability of occurrence or outcome of vaccine risks. Uncertainty is closely related to people's assessment of risk (18). Risk Perception (RP) describes people's attitudes and intuitive judgments about uncertain events in a given situation (19), or people's attitudes and perceptions of risk. The psychometric theory of risk suggests that people can assess risky events in terms of two factors: “fear of risk” and “uncertainty about risk,” with the first factor referring to “uncontrollable things” and the second factor referring to “unfamiliar things” (20). Vaccine hesitant individuals, unfamiliar with vaccine technology (21), are prone to waver between strategies and fail to decide promptly, resulting in a perception of high risk. Social risk amplification theory suggests that risk events interact in various ways with psychological, social, organizational, and cultural factors that may amplify or diminish public reactions to risks or risk events (22). Moreover, the effects of risk events sometimes far exceed the harm of the event itself and can lead to significant indirect effects (23), such as increased RPs of epidemics. Based on this, the following hypothesis is proposed.

**H2:** VH is positively predictive of RP.

The Common Sense Model of RP states that information about health threats activates and develops a risk representation of the disease in question (24). It consists of two components: one is threat/risk perception, and the other is the emotional response. At the level of emotional response, it is represented by emotional states such as tolerance and worry about the potential adverse consequences of uncertain (25). When individuals experience negative emotions such as worry, and this process is repeated over and over again, it leads to the development of anxiety symptoms (14). This means that in uncertain situations, factors such as an individual's concern about possible outcomes and tolerance for event uncertainty can lead to the development of anxiety symptoms (26). In addition, the perceived risk of public emergencies also induces an additional load on individuals and is a key factor in depleting their energy, thereby exerting a negative impact on their work and lives (27). Based on this, the following hypothesis is proposed in this paper.

**H3:** RP is positively predictive of GAD.
**H4:** The effects of VH on GAD will be partially mediated by the RP.

### Mediating role of vaccination status

While the above hypothesis may answer the question of how VH affects generalized anxiety, it does not answer the question of whether the relationship between the two is different under different contexts of vaccination. Does vaccination status have an impact on COVID-19 risk perception? The most important reason for vaccination is self-protection, which reduces the risk of infections to a certain extent (1). But there are also social reasons such as wider economic impact, health equity, moral responsibility, etc. (28), and these benefits of vaccination can reduce the public's perception of COVID-19 risk, affect individual wellbeing, and improves the overall wellbeing of society. Cognitive Dissonance theory suggests that cognitive dissonance occurs when individuals are confronted with new information that contradicts their beliefs or opinions or when they perform actions that go against their personal beliefs or opinions (29, 30). Vaccine-hesitant individuals may develop cognitive dissonance as a result of the fact of being vaccinated or withholding vaccination. Due to the vaccination or non-vaccination facts, individuals who are hesitant about vaccines may have cognitive dissonance. To seek the consistency of the inner world, they either reduce cognitive dissonance by increasing new cognition or avoid increasing the degree of cognitive dissonance by avoiding contacting new cognition (29, 31). After vaccination, individuals may become less hesitant, thereby reducing RP. In addition, vaccination can enhance people's confidence in fighting a pandemic. Therefore, vaccination status may moderate the effects of VH. Based on this, the following hypothesis is proposed in this paper.

**H5:** Vaccination status negatively moderated the relationship between VH and RP, i.e., the positive association between VH and RP was stronger in vaccinated individuals than in unvaccinated individuals.
**H6:** Vaccination status negatively moderated the relationship between VH and GAD, i.e., VH was less positively associated with GAD in vaccinated individuals than in unvaccinated individuals.

**Figure 1:**
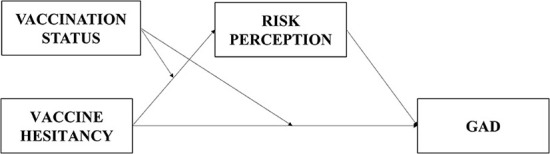
The relationship model.

Based on the above analysis, this paper proposes a mechanism for the effect of VH on GAD through two studies, suggesting that VH affects GAD through RP and that the effect of VH on RP and GAD differs between different vaccination statuses. The relationship model is shown in Figure 1.

## Study one: The relationship between VH and GAD

### Methods

#### Participants

An online questionnaire was administered to Chinese residents between February 4 and 10, 2021. This sampling period was ~1 month after the Chinese government introduced free COVID-19 vaccination (9 January 2021). A total of 811 questionnaires were collected. Invalid questionnaires including those with identical answers and those with an answer time of fewer than 300s were removed. The Mahalanobis distance is used to exclude data outside the 0.001 standards and the SPSS is used to remove abnormal data (32). After the filtering process, a total of 654 valid questionnaires were collected with an effective rate of 80.64%. The age range of the participants was between 16 and 69 years old, with a mean age of 34.27 ± 10.713.

#### Measures

##### General demographics questionnaire

A general questionnaire that contains various demographic variables such as age is used.

##### Vaccine hesitancy

VH was measured by a self-administered questionnaire that includes questions such as, “In general, please rate how hesitant you are about getting vaccinated against COVID-19.” A 5-point scale from “very hesitant” to “not at all” was used in the questionnaire, with higher scores associated with higher levels of vaccine hesitancy.

##### Generalized anxiety disorder

The Generalized Anxiety Disorder-7 (GAD-7) scale, as outlined in “The Diagnostic and Statistical Manual of Mental Disorders,” 5th Edition is an effective evaluation for measuring anxiety symptoms (33). GAD-7 was used to assess the subject's frequency of anxiety symptoms over the past 2 weeks. It consists of 7 questions (e.g., “Feeling afraid that something terrible is going to happen”) on a 4-point scale, the higher the score the more severe the anxiety symptoms. In this study, Cronbach's α coefficient was 0.955.

#### Data analysis

Quantitative data in this study were expressed as means and standard deviations, while qualitative data were expressed as percentages. SPSS 23.0 was used to statistically analyze data.

Pearson correlation analysis and linear regression analysis were used to explore the relationship between VH and GAD.

## Results

### Socio-demographic profile of participants and scores of their vaccine hesitancy

Table 1 shows the sociodemographic profile of participants' gender, education, and vaccination status, as well as scores of their vaccine hesitancy.

**Table 1:** Socio-demographic profile of participants and scores of their VH in study one (*N* = 654).

**Variables**	***n***	**Scores of VH (*M ± SD*)**
**Gender**		
Male	377	2.244 ± 1.134
Female	277	2.567 ± 1.216
**Education**		
Junior high school and below	8	2.625 ± 1.188
High school (junior high school)	110	2.146 ± 1.100
University undergraduate (specialist) and above	536	2.425 ± 1.191
**Vaccination status**		
Yes	354	2.186 ± 1.151
No	300	2.610 ± 1.179

n, Number of participants; VH, Vaccine Hesitancy.

#### Descriptive statistics and correlation analysis

The means, standard deviations, and correlation coefficients for VH and GAD are shown in Table 2. It's evident that VH was negatively correlated with age and positively correlated with GAD.

**Table 2:** Descriptive statistics and correlations for VH and GAD in study one.

**Variables**	**1**	**2**	**3**
Age	1		
VH	−0.139^***^	1	
GAD	0.004	0.328^***^	1
M	34.27	2.38	10.27
SD	10.713	1.179	4.185

*** P < 0.001. VH, Vaccine Hesitancy; GAD, Generalized Anxiety Disorder. The standard for the following table is the same.

#### Linear regression analysis

A structural equation was constructed using GAD as the outcome variable and VH as the predictor variable as shown in Table 3. The results showed that the Durbin-Watson coefficient was 1.994, indicating that there was no significant autocorrelation across subjects in the sample. It also showed that VH positively predicted GAD. This result verified hypothesis 1.

**Table 3:** Regression analysis of VH and GAD.

**Predictor variable**	**Outcome variable: GAD**			
	***B***	***SE***	***P***	**95% CI**
Constant	6.752	0.651	<0.01	[5.474, 8.031]
Age	0.020	0.015	0.174	[−0.009, 0.049]
VH	1.199	0.133	<0.01	[0.937, 1.461]
Joint explanatory power	*R*^2^ = 0.111			
Overall significance	*F*_(1, 644)_ = 40.352^***^			

*** P < 0.001. VH, Vaccine Hesitancy; GAD, Generalized Anxiety Disorder.

## Study two: The influence mechanism of VH on GAD

Study one explained the relationship between VH and GAD and showed that VH positively predicted public GAD during the COVID-19 pandemic, validating the self-discrepancy theory. However, Study one only tested a part of the theoretical model and was unable to explain the mechanism through which VH contributes to GAD. It's not clear whether the cognitive process of VH increases the RP and, in turn, leads to increased anxiety symptoms. Therefore, building on the result of Study one, Study two will clarify the role of RP in the generation of GAD.

### Methods

#### Participants

From 18 February to 4 March 2021, an online questionnaire was administered to Chinese residents using the online survey platform “Questionnaire Star.” A total of 4,301 questionnaires were collected, of which 3,282 were valid with an effective rate of 76.31%. Invalid questionnaires including those with identical answers and those with an answer time of fewer than 300s were removed. The Mahalanobis distance is used to exclude data outside the 0.001 standards and the SPSS is used to remove abnormal data (32). The age range of the participants was between 10 and 81 years old, with a mean age of 33.31 ± 14.591. Among the valid questionnaires, 1,251 of the participants had received the COVID-19 vaccine and 2,031 of them had not received the vaccine.

#### Measures

##### General questionnaire

A general questionnaire was used that contains various demographic variables, such as age, and vaccination status.

##### Vaccine hesitancy

Same as study one.

##### Generalized anxiety disorder

Same as study one and the Cronbach's α was 0.959 in this study.

##### The COVID-19 risk perception

This study employs the COVID-19 Risk Perception Scale developed by Xi et al. (25), which consists of nine questions, including the emotional feeling dimension (e.g., “How likely do I think I will have COVID-19?”), cognitive judgment dimension (e.g., “I am convinced that I will not be sick with COVID-19”), and psychological representation of unusual severity dimension (e.g., “Imagining myself tested positive for COVID-19 is something I find to be _”). Options are scored on a Likert scale of 4 to 6 points. In this study, Cronbach's alpha coefficient was 0.840.

#### Statistics and common method deviation test

Quantitative data in this study were expressed as means and standard deviations, while qualitative data were expressed as percentages. SPSS 23.0 was used to statistically analyze data. Pearson correlation analysis was used to explore the relationship between different variables, and pathway analysis was performed using the PROCESS macro program.

The Harman single factor method was used to detect the common method deviation. The results showed that a total of 2 factors were generated without rotation, with the first factor having a variance interpretation rate of 38.464%, (< 50%) (34), indicating that there was no significant common method bias in this study.

## Results

### Socio-demographic profile of participants and scores of their vaccine hesitancy

Table 4 shows the sociodemographic profile of participants' gender, education, and vaccination status, as well as scores of their vaccine hesitancy. The results showed significantly lower vaccine hesitancy in the vaccinated than in the unvaccinated (*t* = −17.084, *p* < 0.001, *Cohen's d* = 0.619).

**Table 4:** Socio-demographic profile of participants and scores of their VH in study two (*N* = 3,282).

**Variables**	***n***	**Scores of VH (*M ± SD*)**
**Gender**		
Male	1,366	2.324 ± 1.199
Female	1,916	2.579 ± 1.210
**Education**		
Junior high school and below	508	2.490 ± 1.244
High school (junior high school)	1,101	2.607 ± 1.155
University undergraduate (specialist) and above	1,673	2.379 ± 1.231
**Vaccination status**		
Yes	1,251	2.031 ± 1.124
No	2,031	2.745 ± 1.184

#### Descriptive statistics and correlation analysis

The means, standard deviations, and correlation coefficients for age, VH, GAD, RP, and vaccination status are shown in Table 5. VH, GAD, RP, and vaccination status were all correlated. Age was significantly correlated with VH, GAD, RP, and vaccination status.

**Table 5:** Descriptive statistics and correlation analysis for VH, GAD, RP, and vaccination status.

**Variance**	**1**	**2**	**3**	**4**	**5**
1. Age	1				
2. VH	−0.177^***^	1			
3. GAD	0.166^***^	0.273^***^	1		
4. RP	−0.047^**^	0.149^***^	0.241^***^	1	
5. Vaccination status	−0.173^***^	0.286^***^	0.070^***^	−0.004	1
M	33.31	2.47	9.83	20.90	1.62
SD	14.591	1.212	3.963	7.345	0.486

** P < 0.01,

*** P < 0.001. VH, Vaccine Hesitancy; GAD, Generalized Anxiety Disorder; RP, Risk Perception.

#### Moderated mediation analysis

Model 4 (5,000 Bootstrap samples) in the SPSS PROCESS macro program developed by Hayes (35) was used for testing mediating effects. The results showed that with age as control variables, VH significantly and positively predicted RP [*B* = 0.898, *t* = 8.373; *95% CI* = (0.688, 1.109)], with the inclusion of mediating variables, RP significantly and positively predicted GAD [*B* = 0.110, *t* = 12.271; *95% CI* = (0.092, 0.128)], and VH significantly negatively predicted GAD [*B* = 0.722, *t* = 13.070; *95% CI* = (0.614, 0.830)], indicating that RP plays a partial mediating role in the relationship between VH and GAD (see Table 6). Hypotheses 1, 2, 3, and 4 were verified.

**Table 6:** Mediation effect of RP and moderation effect of vaccination status.

**Predictor variable**	**Outcome variable: RP**				**Outcome variable: GAD**			
	***B***	***SE***	***P***	**95% CI**	***B***	***SE***	***P***	**95% CI**
Constant	23.015	0.591	<0.01	[21.856, 24.175]	8.792	0.004	<0.01	[8.074, 9.510]
Age	−0.013	0.009	0.135	[−0.031, 0.004]	−0.033	0.003	<0.01	[−0.042, −0.024]
VH	2.138	0.392	<0.01	[1.370, 2.907]	0.680	0.201	<0.01	[0.286, 1.074]
Vaccination status	−0.954	0.279	<0.01	[−1.502, −0.406]	−0.108	0.143	0.450	[−0.388, 0.172]
Product term	−0.703	0.229	<0.05	[−1.151, −0.254]	0.033	0.117	0.777	[−0.197, 0.263]
RP					0.110	0.009	<0.01	[0.092, 0.127]
Joint explanatory power	*R*^2^ = 0.030^***^				*R*^2^ = 0.129^***^			
Overall significance	*F*_(4, 3228)_ = 24.035				*F*_(5, 3227)_ = 95.844			

*** P < 0.001. VH, Vaccine Hesitancy; GAD, Generalized Anxiety Disorder.

A moderation effect test was conducted using Model 8 in the SPSS PROCESS macro program to examine the moderating role of vaccination status in the first half of the pathway and the direct pathway in the model of VH's impact on GAD through RP. The results are shown in Table 6. VH was not a significant predictor of GAD [*B* = −0.108, *t* = −0.756; *95% CI* = (−0.388, 0.172)] and the interaction term between VH and vaccination status was not a significant predictor of GAD [*B* = 0.033, *t* = 0.283; *95% CI* = (−0.197, 0.263)]. Thus, vaccination status did not play a significant moderating role in the direct pathway by which VH affects GAD. In addition, VH was found to be positively predictive of RP [*B* = −0.954, *t* = −3.416; *95% CI* = (−1.502, −0.406)]. The interaction term for VH and vaccination status was a significant predictor of RP [*B* = −0.703, *t* = −3.068; *95% CI* = (−1.151, −0.254)]. Therefore, the moderating effect of vaccination status was significant.

**Figure 2:**
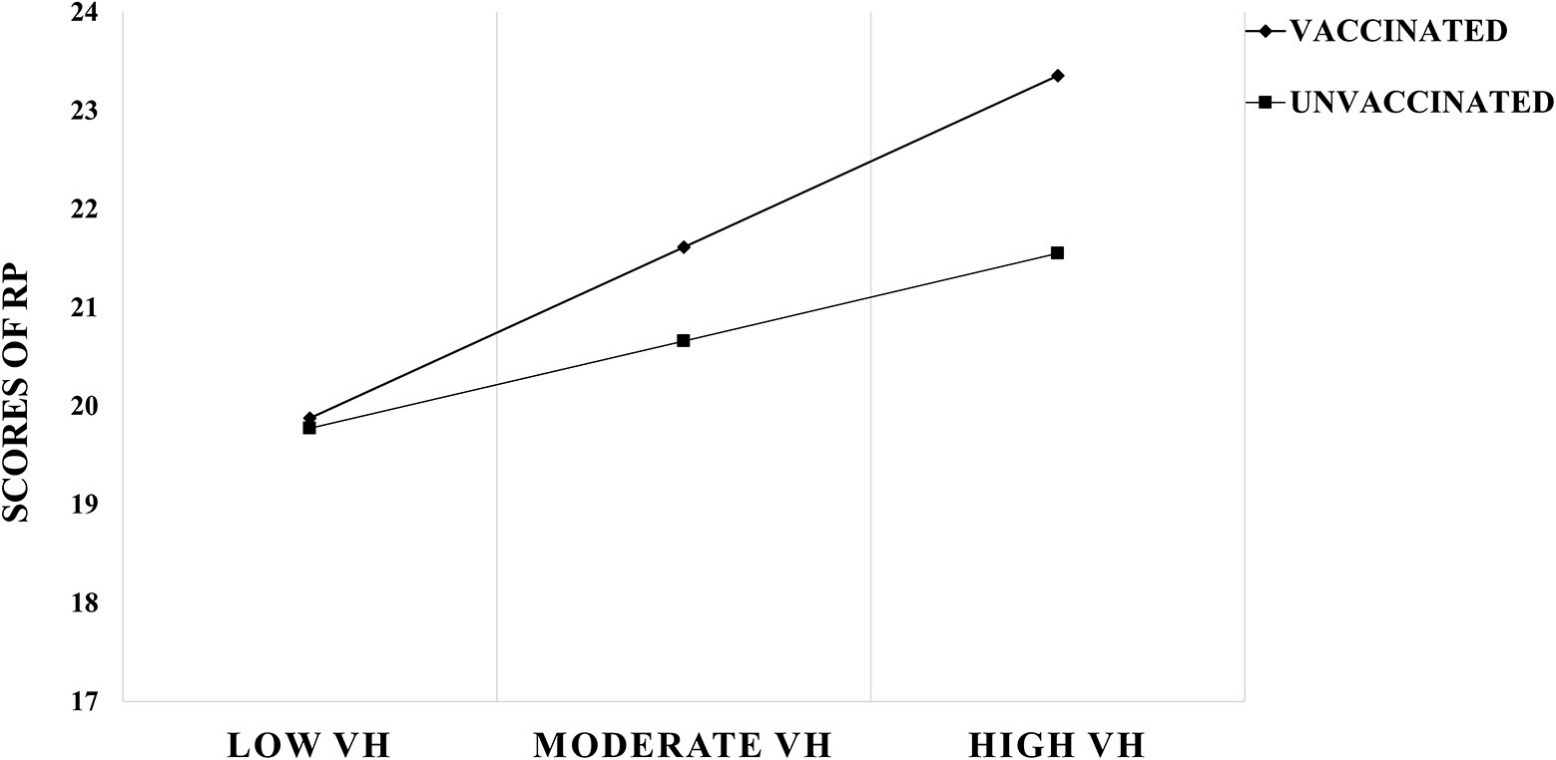
The moderation effect.

Simple slope analysis was conducted to further examine the moderating effect of vaccination status, with groupings based on vaccination or non-vaccination. It was found that VH in the unvaccinated group significantly and positively predicted RP [*B*_*simple*_ = 1.436, *t* = 7.804; *95% CI* = (1.075, 1.797)]; whereas in the vaccinated group, the positive predictive effect became smaller [*B*_*simple*_ = 0.734, *t* = 5.314; *95% CI* = (0.463, 1.004)]. This suggests that the perceived risk of COVID-19 tends to increase with stronger VH, but the trend of change was lower in the unvaccinated group compared to the vaccinated group. The results are shown in Table 6 and Figure 2. Taken together, the moderated mediation model constructed in this study is valid [*Index* = −0.077, *SE* = 0.028, *95% CI* = (−0.134, −0.024)], with vaccination status moderating the first half of the mediating effect, confirming the hypothesis 5 and rejecting hypothesis 6.

For those who have been vaccinated, when their vaccine hesitancy is reduced, it will be easier to reduce risk perception and thus GAD.

## Discussion

### VH positively predicts GAD

Through two cross-sectional studies, it was found that VH was a significant positive predictor of GAD, i.e., as individuals became more hesitant about vaccination, their disorder also increased. This finding is consistent with previous studies (5). The result reveals the predictive effect of VH on anxiety symptoms, thus confirming the self-discrepancy theory (36). Individuals with high VH tend to repeatedly question whether their decision about vaccination is correct. There is a contradiction between the concern about the efficacy and risk of the COVID-19 vaccine (the real self) and the moral and social obligation of vaccination (the should self). The actual-ideal self-discrepancy and the actual-should self-discrepancy a contributing factors to many mental health issues, such as depression, anxiety disorders, and eating disorders (36). They may believe that they will be punished or blamed for their failure to fulfill their responsibility and this negative feeling often leads to anxious moods (36, 37) and even psychiatric disorders. In contrast, individuals with low VH have weaker actual-should self-discrepancy and less associated negative feelings such as anxiety.

### Mediating role of RP

COVID-19 risk perception partially mediates the relationship between VH and GAD. It means that VH can either directly contribute to elevated GAD or indirectly act upon GAD by increasing RP. Vaccine hesitant individuals who are unfamiliar with vaccine technology due to, for example, a lack of comprehensive information (21) tend to rely on intuitive judgments and assessments of risk events, predisposing them to perceptions of high risk. And due to the amplification effect, risk events may amplify or attenuate the public's response to a risk or risk event in a risky situation (23). Because of the amplification effect, VH can lead to high perceptions of COVID-19 risk, leaving individuals in a state of panic. Furthermore, the actual-should self-discrepancy embodied in VH may also exacerbate uncertainty among residents, and hesitancy about vaccine technology may extend to the uncertainty about society as a whole (38). This vicious cycle can then lead to the development of anxiety symptoms (14).

The result reveals a mechanism for the generalization of VH and its effect on psychological risk, i.e., individual's GAD will increase through the amplification of RP. It confirms that VH is not only harmful in the medical sense but also poses a threat to an individual's mental health.

### Moderating role of vaccination status

This study also examined the moderating effect of vaccination status, which played a role in the first half of the mediated pathway, namely the relationship between VH and RP. Vaccination status negatively moderated the relationship between vaccine hesitancy and risk perception, i.e., there was a lower trend of change in the unvaccinated group compared to the vaccinated group. And we found that the vaccine hesitancy was significantly lower in the vaccinated group compared to the unvaccinated group. Therefore, it can be concluded that for the vaccinated group, when their vaccine hesitancy is reduced, it will be easier to reduce the risk perception and thus the GAD. When vaccination becomes a fact, vaccine-hesitant individuals who want to seek consistency between their cognition and behavior tend to mitigate the degree of cognitive dissonance by absorbing new information (29, 30). Therefore, they may be more receptive to interventions such as vaccine science knowledge that affect GAD by intervening in vaccine hesitation. Furthermore, this study found that vaccination status did not moderate the direct pathway of VH on GAD, i.e., VH's direct effect on GAD will not be influenced by the vaccination status. It is evident that vaccination status only moderates VH's effect on GAD when RP is at play. This result further validates the cognitive dissonance theory.

### Inspiration

The emergence of the COVID-19 pandemic has made many determinants of poor mental health more seriously (39). During the COVID-19 pandemic, a meta-analysis shows that there were an additional 762 million cases of anxiety disorders worldwide (a 25.6% increase), with an overall prevalence of 4,802,400 cases per 100,000 population (40). How to deal with the vast amount of anxiety disorders group throughout the COVID-19 pandemic? Our results suggest that vaccine hesitation may be a mediated predictor, and the vaccine status could be an effective moderated factor in the first half of the mediating pathway. And these results enlighten us that the methods of anxiety disorder intervention could be found in a new psycho-social way of vaccine hesitation and vaccination. Vaccinated group has significantly lower vaccine hesitation than the unvaccinated, and furtherly moderates more significantly on risk perception, which could lead to fewer anxiety disorders in some extent. This result also has a positive implication for the promotion of vaccination, i.e., vaccination not only physically has a protective effect on individuals, but also psychologically.

### Limitations and prospect

This study is still a cross-sectional study and can only discuss the correlation between variables, not the causal effect between them. Follow-up studies could be conducted using a longitudinal design to provide more evidence to reveal the causal effect between variables. Because we consider less socio-demographic information when taking data, this results in our inability to effectively identify which individuals exhibit lower vaccine hesitancy. Future studies could further refine the demographic distribution of vaccine hesitancy. In addition, we need to note that vaccine hesitancy exists at all times.

## Conclusion

Vaccine hesitancy predicts generalized anxiety disorder through risk perception, but the mediating role of risk perception is moderated by vaccination status, which means that for the vaccinated group when their vaccine hesitancy is reduced, it will be easier to reduce the risk perception and thus the generalized anxiety disorder. This can be used for the development of interventions to improve mental health and decrease the anxiety disorders through the new psycho-social way of vaccine hesitation and vaccination.

## Data availability statement

The raw data supporting the conclusions of this article will be made available by the authors, without undue reservation.

## Ethics statement

Ethical review and approval was not required for the study on human participants in accordance with the local legislation and institutional requirements. Written informed consent to participate in this study was provided by the participants' legal guardian/next of kin.

## Author contributions

Material preparation, data collection, and analysis were performed by BW, XZ, HF, MH, and RH. The first draft of the manuscript was written by BW and XZ. HF reviewed and revised the manuscript. All authors commented on previous versions of the manuscript and contributed to the study's conception and design, read, and approved the final manuscript.

## Funding

This work was supported by the project fund by the National Natural Science Foundation of China (71704017), Institute of Psychology, Chinese Academy of Science (GJ202003), the Research Foundation of Student Education Management and Reform Research Project funded by Southwest University of Science and Technology (19sxb118), and Sichuan Province Innovation and Entrepreneurship Policy (S202210619064).

## Conflict of interest

The authors declare that the research was conducted in the absence of any commercial or financial relationships that could be construed as a potential conflict of interest.

## Publisher's note

All claims expressed in this article are solely those of the authors and do not necessarily represent those of their affiliated organizations, or those of the publisher, the editors and the reviewers. Any product that may be evaluated in this article, or claim that may be made by its manufacturer, is not guaranteed or endorsed by the publisher.
